# The Seven-parameter Diffusion Model: an Implementation in Stan for Bayesian Analyses

**DOI:** 10.3758/s13428-023-02179-1

**Published:** 2023-08-28

**Authors:** Franziska Henrich, Raphael Hartmann, Valentin Pratz, Andreas Voss, Karl Christoph Klauer

**Affiliations:** 1 Department of Psychology, University of Freiburg, Engelbergerstraße 41, D-79106 Freiburg, Germany; 2 University of Heidelberg, Heidelberg, Germany

**Keywords:** Ratcliff diffusion model, Bayesian inference, Stan function, Model fitting

## Abstract

Diffusion models have been widely used to obtain information about cognitive processes from the analysis of responses and response-time data in two-alternative forced-choice tasks. We present an implementation of the seven-parameter diffusion model, incorporating inter-trial variabilities in drift rate, non-decision time, and relative starting point, in the probabilistic programming language Stan. Stan is a free, open-source software that gives the user much flexibility in defining model properties such as the choice of priors and the model structure in a Bayesian framework. We explain the implementation of the new function and how it is used in Stan. We then evaluate its performance in a simulation study that addresses both parameter recovery and simulation-based calibration. The recovery study shows generally good recovery of the model parameters in line with previous findings. The simulation-based calibration study validates the Bayesian algorithm as implemented in Stan.

Diffusion models (DMs) are among the most frequently used model families in modeling two-alternative forced-choice tasks (see Wagenmakers, 2009, for a review). DMs allow one to model response times and responses in two-alternative forced-choice tasks jointly. In this article, we focus on a seven-parameter version of the model that includes inter-trial variability in several of its components (Ratcliff and Rouder, 1998) as detailed below.

Since its introduction to psychological research, a number of user-friendly software tools have been developed to estimate the model parameters (Vandekerckhove & Tuerlinckx, 2007; Voss & Voss, 2007; Wagenmakers et al., 2007). Bayesian implementations have been proposed for use with WinBUGS (Vandekerckhove et al., 2011), JAGS (Wabersich & Vandekerckhove, 2013), Stan (Carpenter et al., 2017), and as a Python package called HDDM (Wiecki et al., 2013). The purpose of this article is to add to the existing Bayesian implementations, and to overcome limitations of the existing implementations. Specifically, the just-mentioned WinBUGS, JAGS and Stan implementations are limited to a more basic four-parameter version of the DM without inter-trial variabilities, whereas HDDM is limited in the choice of priors that users can specify.

Here, we provide an implementation of the seven-parameter model within the probabilistic programming language Stan (Carpenter et al., 2017). Stan is a free, open-source software that gives the user huge flexibility in defining and varying model properties such as the choice of priors. Stan runs on all major platforms and interfaces with the most popular data analysis languages (R, Python, shell, MATLAB, Julia, Stata).

In the following sections, we first briefly introduce the diffusion model. Following this, we provide details on our new Stan implementation. Finally, we present two sanity checks for our implementation: a simulation study showing good recovery on simulated data, and a simulation-based calibration study analyzing the same simulated data, providing a more rigorous test of the correctness of our algorithm.

**Fig. 1:**
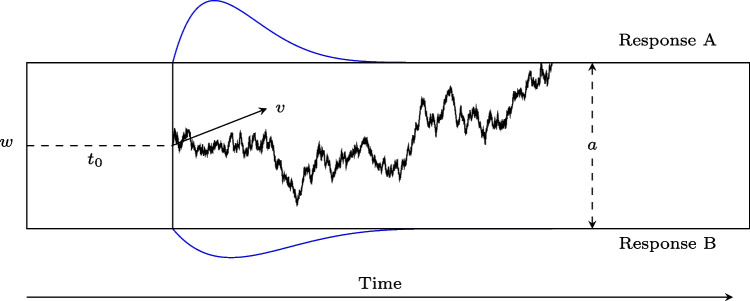
Realization of a four-parameter diffusion process modeling the binary decision process. *Note.* The parameters are the *boundary separation* *a* for two response alternatives, the *relative starting point* *w*, the *drift rate* *v*, and the *non-decision time* $t_0$. The decision process is illustrated as a jagged line between the two boundaries. The predicted distributions of the reaction times are depicted in blue

## The diffusion model

The basic four-parameter DM, first introduced by Ratcliff (1978), is a sequential sampling model used to explain data from two-alternative forced-choice tasks. It has been widely applied to tasks as, for example, the Eriksen flanker task (Assink et al., 2015; Eriksen & Erisken, 1974; White et al., 2010), among many others. In the Eriksen flanker task, participants decide whether a central target arrow among a set of distractor arrows points to the *left* or to the *right* (e.g., $$<<<>
<<<$$).

In diffusion modeling, it is assumed that participants accumulate evidence towards either of two response options on a unidimensional evidence scale on which two boundaries are placed, one for each response option. The distance between both boundaries is denoted as *boundary separation*, *a*. Participants start with a state of evidence placed between the two boundaries on the evidence scale. This point is denoted as *relative starting point*, *w*. Accommodating the possibility of prior bias, this starting point needs not to be equidistant from both boundaries. Participants then accumulate decision-relevant evidence from the environment until a boundary is reached. The evidence-accumulation rate is denoted as *drift rate*, *v*. The evidence accumulation process is noisy and is therefore approximated by a diffusion process. When a boundary is met, a decision for the associated response option is made. All time costs for processes that do not belong to the decision process are summarized in the *non-decision time*, $t_0$. Based on those four parameters (for the basic model), a DM predicts the probability to choose one or the other response alternative and models the distributions of response times associated with each alternative.

In Fig. 1, a diffusion process is depicted. Since the evidence accumulation process is influenced by random noise, the process is drawn as a jagged line. One main advantage of the diffusion model is that the parameters can be interpreted in terms of cognitive processes. For example, the boundary separation is higher when the participant is focused on accuracy, the absolute value of the drift rate is smaller when stimuli are harder to discriminate, the non-decision time is higher for a more time-consuming form of response, and the relative starting point moves towards a decision alternative for which the participant is rewarded (e.g., Arnold et al. , 2015; Lerche and Voss , 2019; Voss et al. , 2004).

According to Ratcliff and Rouder (1998), the basic four-parameter model has problems accounting for the full range of data in two-alternative forced-choice tasks. For example, the model predicts identical reaction time distributions for correct and error responses, if the relative starting point is centered between the boundaries. However, it may occur that, having a centered relative starting point, errors are slower than correct responses. Slow errors can be modeled with inter-trial variability in drift rate, because for a large drift rate, reaction time is short and accuracy is high, whereas for a small drift rate, reaction time is slower and accuracy is lower. In sum, given variability in drift rate, the percentage of slow responses will increase among errors more than among correct responses. Another possibility is that errors are faster than correct responses. This reaction time pattern of fast errors can be modeled with inter-trial variability in starting point, because for a starting point near the correct response boundary, there will be few errors and they will be slow, whereas for a starting point near the error response boundary, there will be more errors and they will be fast. In sum, given variability in starting point, the percentage of fast responses will increase among errors more than among correct responses (Forstmann et al., 2016). For such reasons, Ratcliff and Rouder introduced the seven-parameter DM, which extends the four-parameter model by adding inter-trial variabilities in the drift rate, the non-decision time, and the starting point. Variability in drift rate is assumed to be normally distributed, and the variabilities in non-decision time and starting point are assumed to be uniformly distributed.

Another problem regards parameter recovery. For an accurate parameter recovery large trial numbers are required. Therefore, sometimes participants in a DM study need to work on many trials (sometimes more than 2,000 trials per participant and condition; e.g., Ratcliff and Smith, 2004). This problem can be mitigated by embedding the DM in a Bayesian hierarchical framework (Vandekerckhove et al., 2011), which allows one to calculate reliable and accurate estimates for the parameters of the decision process despite sparse data at the individual level by combining information from both levels, the individual and the group level^1^. This partial pooling yields more robust parameter estimates than does fitting data for each individual separately (Rouder & Lu, 2005). Furthermore, this approach is helpful in integrating data across studies such that one can synthesize the evidence for the overall effects and can analyze how effects changed or did not change across studies (e.g., Pleskac et al., 2018).

Therefore, the next logical step is to combine the seven-parameter model with the Bayesian hierarchical framework. An implementation of the highly efficient Hamiltonian algorithm for Markov chain Monte Carlo estimation (MCMC, Neal, 2011) in the form of the No-U-Turn Sampler (NUTS, Hoffman & Gelman, 2014) is given in Stan. Stan is a probabilistic programming language for statistical modeling and high-performance statistical computation. Stan, named after one of the pioneers in Monte Carlo methods, Stanislav Ulam, provides the user with tools for full Bayesian statistical inference and hierarchical modeling. The MCMC method draws samples from the joint posterior distribution of the parameters of a Bayesian model, which are used to draw inferences on the model parameters and the model fit. Stan is free and open-source, and every user is invited to participate in the development of new features. Users can add new functions by specifying the logarithm of a density (log-density) in the C++ based Stan language (Stan Development Team, 2023a).

## The stan function $\mathtt {wiener\_full\_lpdf()}$

We implemented the log-density of the first-passage time distribution of the seven-parameter DM to provide the new function $\mathtt {wiener\_full\_lpdf()}$ for Stan users. Since we added the function in Stan’s math library (Stan Development Team, 2023b), the function can be used with every interface that supports Stan. The Hamiltonian Monte Carlo algorithm relies on partial derivatives of the log-likelihood function to sample the posterior distribution more efficiently (Neal, 2011). As deriving the derivatives for each model can be cumbersome, Stan automatically computes these partial derivatives using reverse-mode automatic differentiation and numerical approximation (Carpenter et al., 2015). For the underlying distributions that are used to build a model, it can make sense, however, to implement the partial derivatives manually. In the case of a very complex function with known partial derivatives, it is much more efficient and accurate to compute the values of the partial derivatives analytically instead of approximating them numerically. Therefore, we used the work by Hartmann and Klauer (2021), who derived the partial derivatives for the first-passage time distribution in diffusion models, to implement both the log-density of the seven-parameter model and its partial derivatives.

The new function $\mathtt {wiener\_full\_lpdf()}$ returns the logarithm of the first-passage time density function for a diffusion model with up to seven parameters for upper boundary responses. The same function can be used to obtain the log-density for the lower boundary as well (see below). Any combination of fixed and estimated parameters can be specified. In other words, with this implementation it is not only possible to estimate parameters of the full seven-parameter model, but also to estimate restricted models such as the basic four-parameter model, or a five- or six-parameter model, or even a one-parameter model when fixing the other six parameters. For example, it is possible to permit variability in just one or two parameters and to fix the other variabilities to 0, or even to estimate a three-parameter model, when fixing more parameters (e.g., fixing the relative starting point at 0.5).

**Fig. 2:**
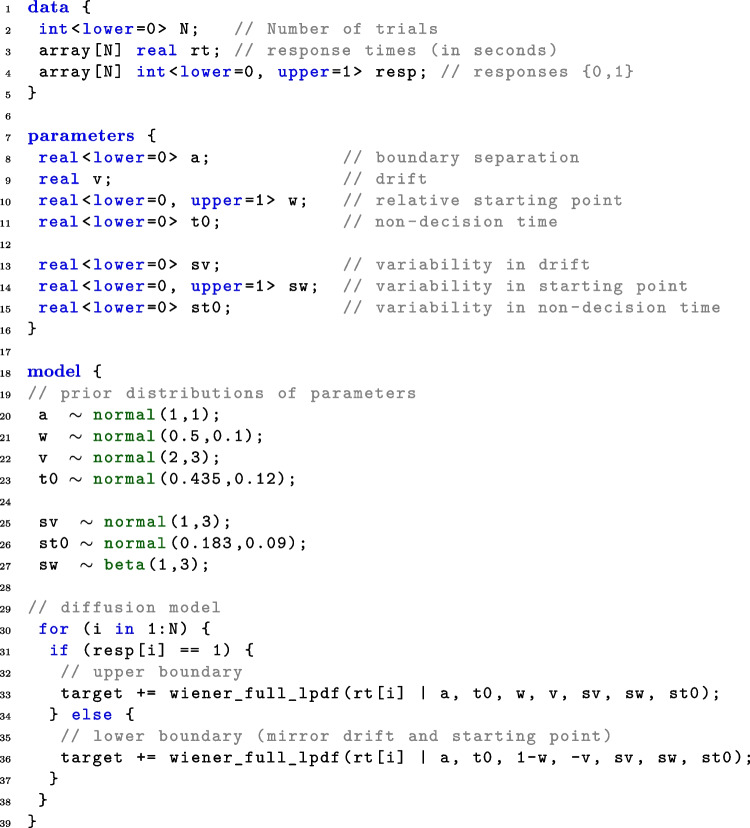
Minimal example of a Stan script for a non-hierarchical seven-parameter DM. *Note.* See text for an explanation of the different components of this script

It is assumed that the reaction time data *y* are distributed according to $\mathtt {wiener\_full()}$:

1 $$\begin{aligned} y \sim \text {wiener\_full}(a,t_0,w,v,s_v,s_w,s_{t_0}). \end{aligned}$$

Mathematically, the function consists of the reaction times, *y*, and the seven parameters, boundary separation, *a*, (lower bound of the) non-decision time, $t_0$, relative starting point, *w*, drift rate, *v*, inter-trial variability of the drift rate, $s_v$, inter-trial variability of the relative starting point, $s_w$, and inter-trial variability of the non-decision time, $s_{t_0}$. It can be stated in the following terms:

2 $$\begin{aligned} {\begin{matrix} &{}\log \bigl [p(y \mid a,t_0,w,v,s_v,s_w,s_{t_0})\bigr ] = \\ &{}\log \bigl [\frac{1}{s_{t_0}}\int _{t_0}^{t_0+s_{t_0}}\frac{1}{s_w}\int _{w-\frac{1}{2}s_w}^{w+\frac{1}{2}s_w}\int _{-\infty }^{\infty } p_3(y-\tau _0\mid a,\nu ,\omega ) \\ &{}\times \frac{1}{\sqrt{2\pi (s_v)^2}}\exp \Bigl (-\frac{(\nu -v)^2}{2(s_v)^2}\Bigr ) \,d\nu \,d\omega \,d\tau _0\bigr ]= \\ &{}\log \bigl [\frac{1}{s_{t_0}}\int _{t_0}^{t_0+s_{t_0}}\frac{1}{s_w}\int _{w-\frac{1}{2}s_w}^{w+\frac{1}{2}s_w} M\times p_3(y-\tau _0\mid a,\nu ,\omega ) \,d\omega \,d\tau _0\bigr ], \end{matrix}} \end{aligned}$$

where *p*() denotes the density function, and *M* and $p_3()$ are defined, by using $t:=y-\tau _0$, as

3 $$\begin{aligned} M := \frac{1}{\sqrt{1+s_v^2t}}\exp \Bigl (av\omega +\frac{v^2t}{2}+\frac{s_v^2a^2\omega ^2-2av\omega -v^2t}{2(1+s_v^2t)}\Bigr )\text {and} \end{aligned}$$

4 $$\begin{aligned} p_3(t\mid a,v,w) := \frac{1}{a^2}\exp \Bigl (-avw-\frac{v^2t}{2}\Bigr )f(\frac{t}{a^2}\mid 0,1,w), \end{aligned}$$

where $f(t^*=\frac{t}{a^2}\mid 0,1,w)$ can be specified in two ways:

5 $$\begin{aligned} f_l(t^*\mid 0,1,w) = \sum _{k=1}^\infty k\pi \exp \Bigl (-\frac{k^2\pi ^2t^*}{2}\Bigr )\sin (k\pi w)\text { and} \end{aligned}$$

6 $$\begin{aligned} f_s(t^*\mid 0,1,w) = \sum _{k=-\infty }^\infty \frac{1}{\sqrt{2\pi (t^*)^3}}(w+2k) \exp \Bigl (-\frac{(w+2k)^2}{2t^*}\Bigr ). \end{aligned}$$

Which of these is used in the computations depends on which expression requires the smaller number of components *k* to guarantee a pre-specified precision (Blurton et al., 2017; Gondan et al., 2014; Hartmann and Klauer, 2021; Navarro & Fuss, 2009).

### How to use the function in Stan

After the mathematical formulation of the seven-parameter diffusion model, we now present a hands-on description of how to use the new function. In the declaration of a Stan model, $\mathtt {wiener\_full}$ can be called in two different ways

$$\mathtt {y \sim wiener\_full(a,t0,w,v,sv,sw,st0);}$$

or

$$\mathtt {target \,\, += wiener\_full\_lpdf(y|a,t0,w,v,}$$

$$\mathtt {sv,sw,st0);}$$

Since the function is not vectorized, it is called for each experimental trial in a for-loop for the reaction time and response observed in the trial with parameters appropriate to the condition (see Fig. 2 for a template). Note that the function always returns the value for the upper response boundary. To compute the value for the lower response boundary the function has to be called with $-v$ instead of *v*, and $1-w$ instead of *w*. The model block shown in Fig. 2 provides a template for calling the function for both, the upper and the lower response boundary.

As pointed out above, $\mathtt {wiener\_full\_lpdf()}$ also allows one to compute restricted models involving one, two, three, four, five, or six parameters by setting parameters to zero or fixing parameters to other given values. For example, $s_v$, $s_w$, and/or $s_{t_0}$ can be set to zero, indicating, in order, no inter-trial variability in *v*, no inter-trial variability in *w*, and/or no inter-trial variability in $t_0$, respectively. Often it might also be useful to set the relative starting point to 0.5 (e.g., when assuming an unbiased decision maker). For example, if no inter-trial variabilities for the relative starting point and for the non-decision time are needed, the function call might look as follows:

target += wiener_full_lpdf(y|a,t0,w,v,

sv,0,0);

For a very parsimonious three-parameter model, assuming no inter-trial variabilities at all and fixing the relative staring point at 0.5, the function call might look as follows:

target += wiener_full_lpdf(y|a,t0,0.5,

v,0,0,0);

It is also possible to control the precision in the computation of the DM partial derivatives^2^ by calling the function $\mathtt {wiener\_full\_prec\_lpdf()}$, analogously:

target += wiener_full_prec_lpdf(y|a,t0

,w,v,sv,sw,st0,precision);

The usage and behavior of the two functions are the same except for the added control over the precision parameter.

### Declaration of the Stan model

To declare a Stan model the user should specify three blocks: the data block, the parameters block, and the model block. In the following, the blocks will be described in some detail (see Fig. 2 for an example of a model declaration for a seven-parameter DM).

#### The data block

The data should consist of at least three variables:

1. The number of trials *N*,
2. the response, coded as $0=$ “lower bound” and $1=$ “upper bound” (in Fig. 2), and
3. the reaction times in seconds (not milliseconds).

Note that two different ways of coding responses are commonly used: First, in *response coding*, the boundaries correspond to the two response alternatives. Second, in *accuracy coding*, the boundaries correspond to correct (upper bound) and wrong (lower bound) responses.

Depending on the experimental design, one would typically also provide the number of conditions and the condition associated with each trial as a vector. In a hierarchical setting, the data block would also specify the number of participants and the participant associated with each trial as a vector. It is also possible to hand over a precision value in the data block.

**Table 1:** Parameter Ranges

Parameter	Range	Parameter	Range
*a*	$(0, \infty )$	*y*^a^	$(0, \infty )$
*v*	$(-\infty ,\infty )$	$s_v$	$[0, \infty )$
*w*	(0, 1)	$s_w$	[0, 1)
$t_0$	$[0, \infty )$	$s_{t_0}$	$[0, \infty )$

^a^ The reaction time, *y*, is not a parameter

#### The parameters block

The model arguments of the $\mathtt {wiener\_full\_lpdf()}$ function that are not fixed to a certain value are defined as parameters in the parameters block. In this block, it is also possible to insert restrictions on the parameters. Note that the MCMC algorithm iteratively searches for the next parameter set. If the suggested sample falls outside the internally defined parameter ranges, the program will throw an error, which causes the algorithm to restart the current iteration. Since this slows down the sampling process, it is advisable to include the parameter ranges in the definition of the parameters in the parameters block to improve the sampling process (see Table 1 for the parameter ranges) as exemplified in Fig. 2. In addition, the parameter space is further constrained by the following conditions:

1. The non-decision time has to be smaller or equal to the RT: $t_0\le y$.
2. The varying relative starting point has to be in the interval (0, 1) and thus,
  7 $$\begin{aligned} {\begin{matrix} &{}w+\frac{s_w}{2}< 1, \text { and} \\ &{}0 < w-\frac{s_w}{2}. \end{matrix}} \end{aligned}$$

#### The model block

In the model block, the priors are defined and the likelihood is called for the upper and the lower response boundary. Different kinds of priors can be specified here. When no prior is specified for a parameter, Stan uses default priors with specifications +uniform(-infinity, infinity)+. For further information see the Stan Development Team (2022b). Generally, mildly informative priors might help to get the full benefit of a Bayesian analysis.

**Fig. 3:**
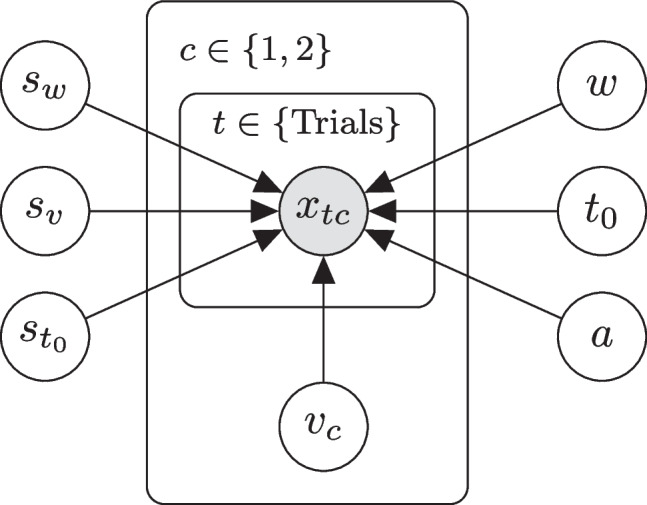
Graphical model representation in the simulation study. *Note.* Each data point $x_{tc}$ (vector of reaction time and response) within trial *t* and condition *c* depends on the seven diffusion parameters, from which only the drift rate varies between conditions. This results in eight parameters to estimate

In the second part of the model block, the likelihood function is applied to all responses. As explained above, this has to be done in a for-loop, and drift rate and relative starting point have to be mirrored for responses at the lower boundary.

## Validating the new function

In this section, we report results from two sanity checks: First, we present a simulation study to test whether our implementation of the full diffusion model is able to recover given parameters and, second, we perform a simulation-based calibration study (Talts et al., 2018) analyzing the same simulated data to test the adequacy of the resulting posterior distributions. For these studies, we chose prior distributions for all parameters as recommended in the literature, sampled different sets of parameters from these distributions, then simulated data from these parameters and ran the model on the data with the same distributions for the priors in order to analyze the results in two different ways.

**Table 2:** Priors for simulation study

Parameter	Prior distribution
*a*	$\mathcal {N}(1,1) \text { T}[0.5,3]$
*v*	$\mathcal {N}(2,3) \text { T}[0,5]$
*w*	$\mathcal {N}(0.5,0.1) \text { T}[0.3,0.7]$
$t_0$	$\mathcal {N}(0.435,0.12) \text { T}[0.2,1]$
$s_v$	$\mathcal {N}(1,3) \text { T}[0,3]$
$s_w$	$\mathcal {B}(1,3)$
$s_{t_0}$	$\mathcal {N}(0.183,0.09) \text { T}[0,0.5]$

*Note.* $\mathcal {N}$ = normal distribution; $\mathcal {B}$ = beta distribution; $\text {T}[.,.]$ = truncation

### Simulation study

We conducted a simulation study to test, on the one hand, the precision of parameter recovery (recovery study), and, on the other hand, whether the new implementation is correct (simulation-based calibration study). For this purpose, we simulated data once and then analyzed these data with respect to both aspects. Simulated datasets comprise trials from two conditions, representing two different stimulus types, where for Condition 1 and 2 positive and negative *drift rates*, respectively, are assumed. All other parameters are shared across conditions as depicted in the graphical model representation in Fig. 3. This is a common design in many reaction time experiments (e.g., see Arnold et al. , 2015; Johnson et al. , 2020; Ratcliff and Smith , 2004; Voss et al. , 2004).

The data were fitted with the full diffusion model, comprising a total of eight parameters (because of the two drift rates). Separate models were fitted for each simulated dataset.

#### Ground truth and priors

The parameters for the simulation, denoted as the *ground truth*, are randomly drawn from the prior distributions used in the model. This is a natural choice for informative priors in the case that the generating model is known, and a prerequisite for the simulation-based calibration.

The parameters are drawn from the distributions shown in Table 2, where $\mathcal {N}$ denotes the normal distribution, $\mathcal {B}$ the beta distribution, and $\text {T}[.,.]$ denotes a truncation. At the same time, these distributions serve as priors in the Stan model. The prior distributions for *a*, *w*, and $s_v$ are based on Wiecki et al. (2013, Fig. 1 in the Supplements), the distributions for $t_0$ and $s_{t_0}$ are based on Matzke and Wagenmakers (2009, Table 3) and the distributions for *v* and $s_w$ are the ones used in Wiecki et al. (2013). To simulate the above-mentioned two conditions, *v* is drawn twice, and the second value is multiplied with the factor $-1$, such that in the first condition, *v* is directed to the upper boundary and in the second condition, *v* is directed to the lower boundary.

**Table 3:** Parameter recovery study: Evaluation criteria (Correlations, Coverage, mMCSE), for parameters estimated from 100, and 500 simulated trials, respectively

Par.	r	$50\%^{\text {a}}$	$95\%^{\text {a}}$	mMCSE^b^
— 100 Trials —				
*a*	.96	51	95	0.00486
$v_1$	.91	48	95	0.01796
$v_2$	.91	50	95	0.01821
$t_0$	.98	49	94	0.00063
*w*	.87	50	95	0.00120
$s_v$	.68	48	94	0.01964
$s_w$	.40	51	95	0.00564
$s_{t_0}$	.83	47	96	0.00133
— 500 Trials —				
*a*	.99	49	94	0.00280
$v_1$	.97	49	95	0.01170
$v_2$	.97	49	94	0.01170
$t_0$	.99	49	94	0.00037
*w*	.97	50	94	0.00063
$s_v$	.84	50	94	0.01486
$s_w$	.62	50	95	0.00543
$s_{t_0}$	.95	49	95	0.00071

*Note.* Par.=Parameters; r=Correlations (between true parameter values and posterior medians)

^a^ Percent of simulated datasets with true value in the HDI of this percentage

^b^ Mean of Monte Carlo standard error (mMCSE) across simulated datasets

#### Datasets

For the choice of the number of datasets we follow the settings used in previous recovery analyses and simulation-based calibration analyses (using between $N=500$ and $N=10.000$ simulated datasets, see Hartmann et al. , 2020; Heck et al. , 2018; Klauer and Kellen , 2018; Lerche et al. , 2017; Talts et al. , 2018; Wabersich & Vandekerckhove , 2013), and consider computational time. Hence, we drew 2000 ground truths from the prior distributions shown in Table 2 and simulated two datasets for each ground truth, resulting in $2\times N=2\times 2000$ datasets.

The first 2000 datasets each consist of 100 simulated trials (50 per condition), and the second 2000 datasets each consist of 500 simulated trials (250 per condition). Many more trials seem to be unrealistic in reaction time tasks and are very costly in terms of computation time. Many fewer trials are assumed to be too few for the successful estimation of inter-trial variabilities (Boehm et al., 2018). Therefore, using 500 trials seemed to be a good compromise, and 100 trials are chosen to see whether the method still performs reasonably well with fewer trials. Data were simulated with the +rdiffusion()+-function of the R-package +rtdists+ (R Core Team, 2021; Singmann et al., 2022) with a precision of 4 and the *fastdm*-method (Voss & Voss, 2007).

#### Method configuration

Analyses were run on the bwUniCluster within the framework program bwHPC with parallelization only in the Stan-model via the $\mathtt {reduce\_sum()}$ routine. We chose to run four chains (as recommended by Vehtari et al., 2021, page 4). All chains were computed sequentially. In calibration studies we found that reducing the maximum treedepth to 5 speeds up the sampling process, while still resulting in good convergence and no divergent transitions.

We started computations with 150 warmup and 500 sampling iterations per chain and repeated computations up to seven times with increased warmup iterations for those datasets for which the model did not converge satisfactorily. For all other method parameters, the Stan default values were taken.

#### Recovery study

##### Convergence and diagnostics

The Stan developers recommend that some diagnostics need to fulfill certain criteria before going deeper into the analysis (e.g., Vehtari et al., 2021). Among these diagnostics are the rank-normalized effective sample size, $N_{\text {eff}}$, the convergence parameter, $\hat{R}$, and the number of divergent transitions.

First, the effective sample size captures how many independent draws contain the same amount of information as the dependent draws obtained by the MCMC algorithm. It is recommended to check that the rank-normalized effective sample size is greater than 400, $N_{\text {eff}}>400$ (Vehtari et al., 2021). A useful heuristic is to ensure that the relative effective sample size is large enough: $N_{\text {eff}}/N_{\text {samp}}>0.1$, where $N_{\text {samp}}$ is the number of samples drawn and retained from the posterior distribution (Stan Development Team, 2022a).

Second, the $\hat{R}$ value is a measure of convergence. This is recommended to be smaller than 1.01, $\hat{R}<1.01$ (Vehtari et al., 2021), due to experience of the authors in practical use. This threshold is much tighter than the value of $\hat{R}<1.1$ first recommended by Brooks and Gelman (1998).

**Fig. 4:**
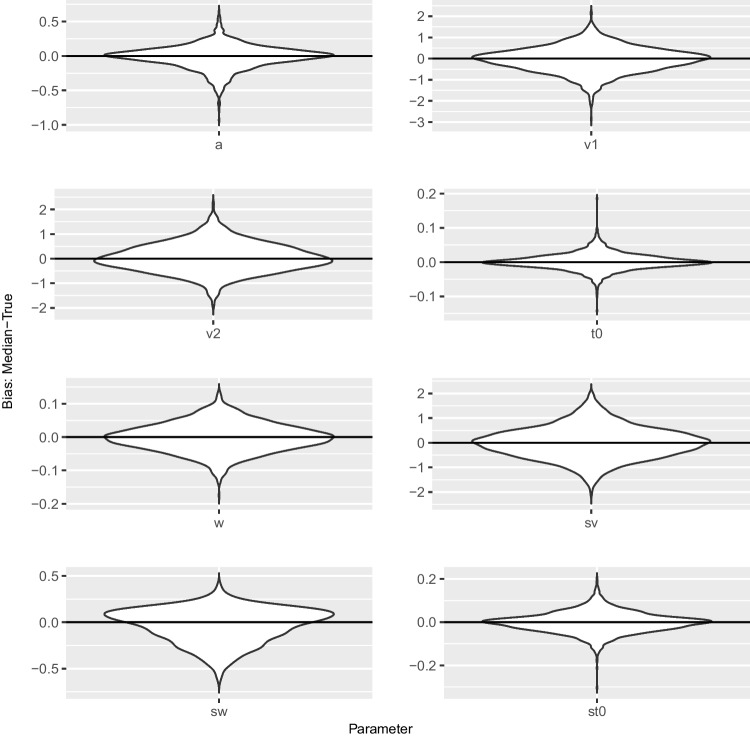
Violin plots of bias between posterior median and true value for 100 trials

Third, there should not be divergent transitions in the sampling process. Divergent transitions can bias the obtained estimates and are an indicator of convergence problems (Vehtari et al., 2021).

Therefore, we checked these diagnostics ($N_{\text {eff}}/N_{\text {samp}}>0.1$, $\hat{R}<1.01$, and divergent transitions), and reanalyzed datasets with insufficient diagnostics with more warmup iterations to ensure the chains have converged at the start of sampling. We started analyses with 150 warmup iterations per chain. As this quickly turned out to be too low to reach the strict convergence criteria, we continued the analyses with higher warmup and sampling iterations per chain. In the end, most of the datasets met the criteria with 1000 warmup and 1000 sampling iterations per chain (about 99%). There were only a few datasets that needed up to 3000 warmup and 1000 sampling iterations (about 1%).

For the retained 4000 MCMC samples, all effective sample sizes are above 400, all relative effective sample sizes are greater than 0.1, nearly all $\hat{R}$ values are smaller than 1.01 (2 out of 32000 $\hat{R}$ were bigger than 1.01) and no divergent transitions occurred. There is one dataset for which the $\hat{R}$ equals 1.012 for the *a* parameter and $\hat{R}$ equals 1.013 for the $s_v$ parameter. But, as these values are still below 1.05 we stopped reanalyzing and included this dataset into further analyses.

**Fig. 5:**
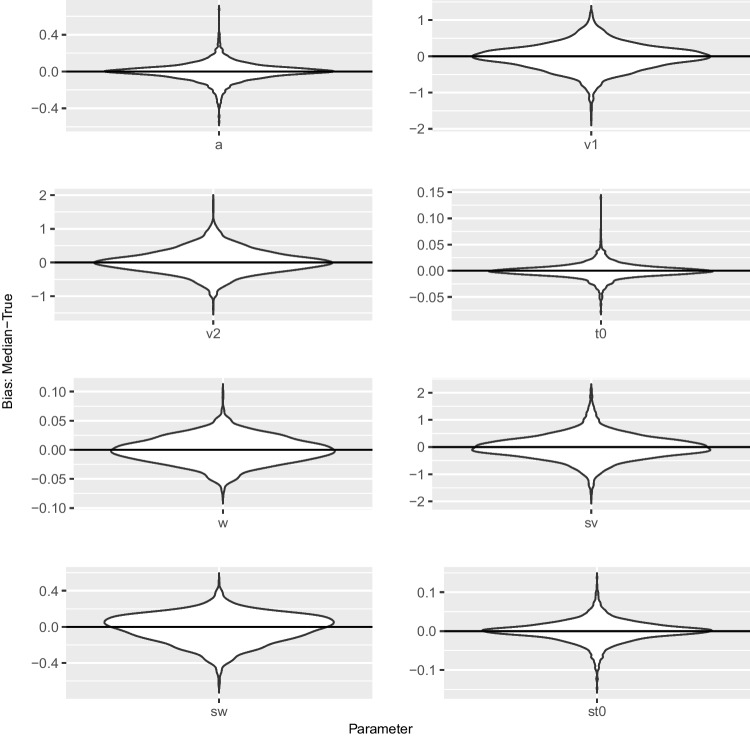
Violin plots of bias between posterior median and true value for 500 trials

##### Recovery

Next, we compute some typical measures to test recovery in the Bayesian context. We present *correlations* between the true values and the posterior medians, *coverage* via the percentage of times across the datasets that the true value lies in the 50% and 95% highest density interval (HDI), respectively, as another measure of recovery, and the mean of the *Monte Carlo standard errors* (mMCSE) as a quantitative suggestion of how big the estimation noise in Markov chains is. The MCSE indicates the estimated SD of the posterior mean in the chain, where SD is the standard deviation of the posterior samples, and is interpreted on the scale of the parameter value (Kruschke, 2015; Vehtari et al., 2021). The MCSE is basically defined as $\text {SD}/\sqrt{N_{\text {eff}}}$. Results are shown in Table 3. Furthermore, we display the *bias* in terms of the difference between the posterior median and the true value in violin plots in Fig. 4 and in Fig. 5 for the datasets with 100 and 500 trials, respectively. Note the different scaling of the *y*-axes in the two figures. In Appendix A, we present a *runtime analysis*.

The correlations show a similar pattern as already found in literature (e.g., Boehm et al., 2018): the three inter-trial variabilities show smaller correlations with the true values than the other model parameters. Nonetheless, in the analyses with 500 trials, correlations of .62 for $s_w$, .84 for $s_v$, and even .95 for $s_{t_0}$ were obtained.

The coverage values meet the expectations in this setup with values between 49% and 50% for 500 trials in 50% HDI and between 94% and 95% for 500 trials in 95% HDI. The MCSE values show that the parameters are estimated with small standard errors that decrease with the number of trials.

MCSE quantifies the variability of parameter estimates calculated from the sample of the posterior distribution, whereas bias assesses systematic deviation of such estimates from the ground truth. The violin plots for 500 trials (Fig. 5) show smaller biases than the violin plots for 100 trials (Fig. 4). All plots except the plot for $s_w$ have most of their mass at 0 and are quite symmetric, meaning that there is no sign of systematic over- or underestimation. The plots for $s_w$ show small overestimation and a non-symmetric distribution of bias. This may reflect the non-symmetric prior distribution for $s_w$. The plots for $v_1$, $v_2$, and $s_v$ show a relatively wide spread, whereas the plots for *w*, $t_0$, and $s_{t_0}$ suggest that these parameters can be recovered with small absolute biases.

In summary, the results of the recovery study are in line with findings in the literature (e.g., Boehm et al., 2018). Specifically, the estimation of the inter-trial variability in relative starting point seems to be tricky in this setup. Nevertheless, results suggest that the new implementation is able to recover the parameters of the seven-parameter diffusion model. As expected, the parameter recoveries based on 500 trials are better than those based on 100 trials, that is, correlations are higher, coverage is better, and mean MCSEs and biases are smaller.

**Fig. 6:**
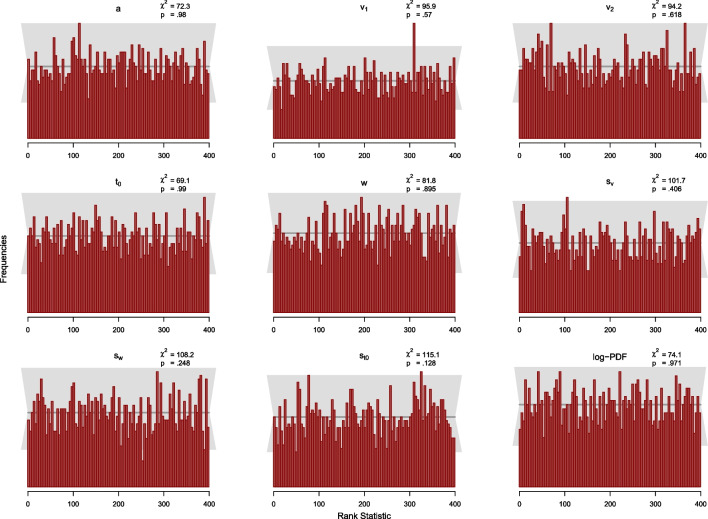
Histograms of the rank statistic for 100 trials. *Note.* The histograms indicate no issues as the empirical rank statistics (*red*) are consistent with the variation expected of a uniform histogram (*gray*)

**Fig. 7:**
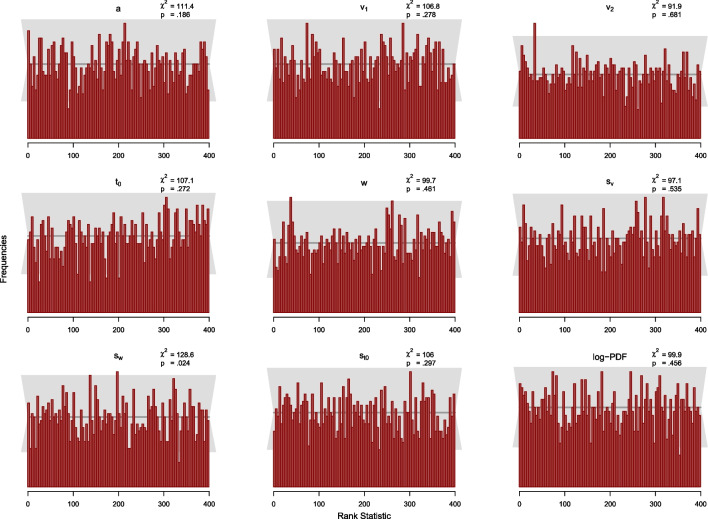
Histograms of the rank statistic for 500 trials. *Note.* The histograms indicate no issues as the empirical rank statistics (*red*) are consistent with the variation expected of a uniform histogram (*gray*)

#### Simulation-based calibration study

Recovery studies in Bayesian contexts are limited by the facts that it is difficult to conclude that a Bayesian algorithm is validly implemented from successful recovery and conversely that it is difficult to conclude that it is invalid from the occurrence of systematic bias (Talts et al., 2018). This is not true for simulation-based calibration (SBC, Talts et al., 2018), which tests whether the algorithm satisfies a consistency condition that a valid algorithm must respect. SBC can therefore reveal conclusive evidence for the invalidity of an invalid algorithm. To further illustrate the correct functioning of the new implementation of the seven-parameter diffusion model, we therefore performed a simulation-based calibration study.

SBC is a method to validate inferences from Bayesian algorithms that generate posterior samples. The method identifies inaccurate computations and inconsistencies in the implementation of the model. According to Talts et al. (2018), the only assumption for SBC is that there exists a generative model for the data. The procedure is to repeatedly sample parameters from the prior distributions, simulate data from these parameters, and fit the model to these datasets using the same priors from which the parameters were sampled. The analysis, if implemented correctly, must satisfy the following *self-consistency condition*:

8 $$\begin{aligned} \pi (\theta ) = \int \int \pi (\theta \mid \tilde{y})\pi (\tilde{y}\mid \tilde{\theta })\pi (\tilde{\theta })\,d\tilde{y}\,d\tilde{\theta }, \end{aligned}$$

where $\tilde{\theta }\sim \pi (\theta )$ are the parameters - denoted as the *ground truth* - sampled from the prior distribution, $\tilde{y}\sim \pi (y\mid \tilde{\theta })$ are the data generated from the model using the ground truth, and $\theta \sim \pi (\theta \mid \tilde{y})$ the posterior samples. This condition implies that the prior sample $\tilde{\theta }$ and the posterior sample $\theta$ follow the same distribution. Modrak et al. (2022) proposed an extension of the SBC check such that the implication not only holds for the parameter space but also includes the data space. From this extension follows that the *rank statistic* $r_{\text {total}}$ of the prior sample relative to the posterior sample, defined for any one-dimensional random variable with domain parameter and data space, $f:\Theta \times Y\rightarrow \mathbb {R}$,

 

9 $$\begin{aligned} \begin{aligned} r_{\text {less}}\left( \{f(\theta _1, y),\dots ,f(\theta _L, y)\}\right)&:= \sum _{l=1}^L{\mathbb {I}\left[ f(\theta _l, y)<f(\tilde{\theta }, y)\right] }\in [0,L]\\ r_{\text {equals}}\left( \{f(\theta _1, y),\dots ,f(\theta _L, y)\}\right)&:= \sum _{l=1}^L{\mathbb {I}\left[ f(\theta _l, y)=f(\tilde{\theta }, y)\right] }\in [0,L]\\ K&\sim \text {uniform}(0, r_{\text {equals}})\\ r_{\text {total}}&:= r_{\text {less}} + K, \end{aligned} \end{aligned}$$

should be uniformly distributed over the natural numbers in [0, *L*], where *L* is the number of samples of the posterior distribution, and $\mathbb {I}$ is the indicator function taking the value 1 if the condition in the parentheses holds and the value 0 otherwise.

Our simulation study was designed to allow us to test this expectation. That is, given a correct implementation of the function $\mathtt {wiener\_full\_lpdf()}$, the SBC should result in uniformly distributed rank values. Specifically, for each model parameter and for the log-density, we compute the rank statistic of the ground truth in the posterior sample, and the histogram of rank statistics as proposed by Modrak et al. (2022). The histogram should reveal a uniform distribution of the rank statistic if the algorithm is valid, whereas systematic deviations from the uniform distribution allow one to diagnose specific problems of the algorithm (Talts et al., 2018).

Since the MCMC-algorithm used in Stan produces autocorrelated samples, we have to thin our posterior samples to obtain (a smaller number of) independent draws from the posterior distribution. As mentioned above, we ensured that all effective sample sizes are above 400. Therefore, we uniformly thin the posterior samples to $$L=399$$ high-quality draws according to Algorithm 2 by Talts et al. (2018), and compute the rank statistic as defined in Eq. 9 for each of the *N* datasets and analyze the resulting histogram for uniformity. For the histograms, we set the number of bins to 100, so that across the 2000 simulated datasets, there are 20 observations expected per bin. In Figs. 6 and 7, we add a gray band to the histogram that covers 99% of the variation expected for each frequency in a histogram of a uniform distribution. Specifically, the band covers the interval from the 0.005 percentile to the 0.995 percentile of the Binomial(*N*; $$100^{-1})$$ distribution.

Additionally, we calculate the $\chi ^2$-statistic for the differences between observed and expected frequencies of observations per bin for each parameter and for the log-density with expected frequencies given by the expected uniform distribution (i.e., 20 per bin). In this way, it is possible to check whether the SBC assumption is significantly violated, which would indicate that the posterior distributions are flawed. For each parameter, the observed $\chi ^2$ value is compared to the critical $\chi ^2$ value of 123.23, for $p=.95$ with $df=99$ (number of bins minus 1). In sum, we calculate 18 $\chi ^2$-statistics, 9 for the 100 trials study and 9 for the 500 trials study. In order to check whether the aggregate of the individual $\chi ^2$-tests follows the hypothesis of uniformity, we also aggregated the resulting 18 *p*-values by means of Fisher’s combined probability test (Fisher, 1950)^3^.

##### Results and Discussion

The results of the SBC study for 100 trials are displayed in Fig. 6 and for 500 trials in Fig. 7. Visual inspection suggests that none of the histograms shows systematic deviation from the uniform distribution. This means that there is no clear pattern in the histograms that indicates some kind of bias as described in Modrak et al. (2022) and Talts et al. (2018).

Furthermore, the $\chi ^2$-statistic testing for uniformity is significant at the 5% level for only one out of 18 calculated statistics: the $\chi ^2$ value for $s_w$ in the simulation with 500 trials was $\chi ^2(99) = 128.6$ with $p =.024$. Note that with 18 tests at the 5% level, one significant result is well within the range of expectations. This is confirmed by Fisher’s combined probability test. Across all 18 *p*-values, the combined test yielded a $\chi ^2(36)$ of 34.65 with $p =.467$, indicating that the set of *p*-values is consistent with the composite hypothesis that all histograms follow a uniform distribution. Taken together, we conclude that there is little indication in these analyses that our DM algorithm is implemented incorrectly.

## General discussion

The purpose of this paper was to introduce a new implementation of the seven-parameter diffusion model in a Bayesian framework – the probabilistic programming language Stan. As mentioned in the introduction, this implementation overcomes a number of shortcomings of previous implementations of the DM. Unlike previous implementations in WinBUGS, JAGS, and Stan, the current implementation enables users to incorporate the variability parameters that define the seven-parameter version of the DM. Unlike the implementation via HDDM, the Stan framework provides the user with great flexibility in choosing priors and in implementing complex hierarchically structured models. Additionally, the new implementation within the Stan framework allows users to benefit from all resources that are available for this platform, including libraries for model comparison (e.g., using loo), or (graphical) analyses of MCMC convergence. In the present paper, we described how to use the newly implemented Stan function and presented simulation studies that examined the recovery of the parameters and a correctness check of the implemented algorithm.

In summary, the results of the recovery study are in line with findings in the literature. We found satisfactory to good parameter recovery in terms of correlations, bias and coverage, with better recovery for the basic model parameters than for the variability parameters as previously observed (e.g., Boehm et al., 2018). Specifically, recovery of the inter-trial variability in relative starting point seems to be tricky in this setup. Nevertheless, simulation-based calibration does not show any systematic errors, suggesting that the implementation is correct and that bias in the estimation reflects the influence of the chosen prior. Furthermore, the results of the simulation-based calibration study suggest that the new algorithm is implemented correctly, and Stan is suitable for fitting DMs with its Hamiltonian MCMC algorithm.

**Fig. 8:**
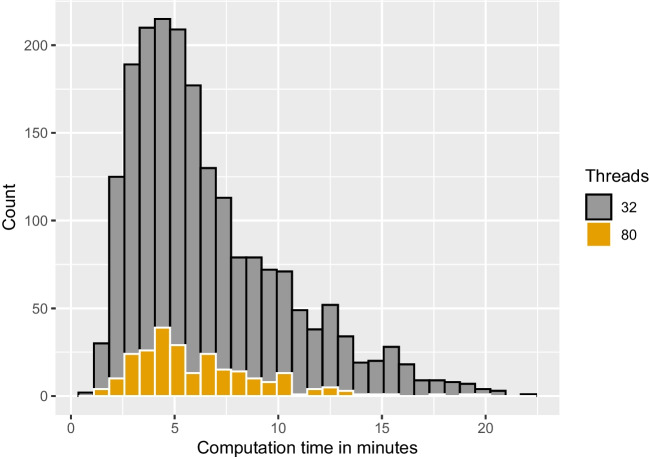
Histograms of runtimes for 100 trials. *Note.* Analyses were parallelized on 32, and 80 threads per chain, respectively. Mean runtime on 32 threads per chain is 6.7 minutes. Mean runtime on 80 threads per chain is 6.2 minutes

**Fig. 9:**
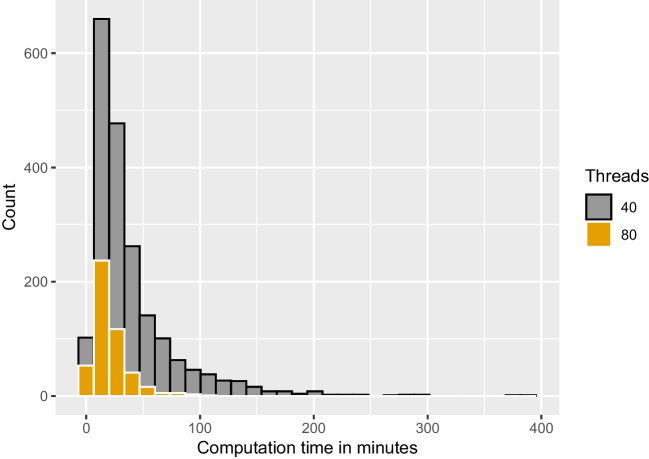
Histograms of runtimes for 500 trials. *Note.* Analyses were parallelized on 40, and 80 threads per chain, respectively. Mean runtime on 40 threads per chain is 44 minutes. Mean runtime on 80 threads per chain is 20 minutes

The design of our simulation studies was constrained by the goals that we pursued in this brief article, namely to implement a number of validity checks of our algorithm. For this reason, the scope of this simulation study is limited to the case with informative priors in a simple non-hierarchical model with data that were generated from the DM model without contaminants as occur in real data. It is thus up to future research to examine the performance of the new Stan implementation in other settings; for example, with less informative or even uninformative priors, with real data, with a hierarchical approach, or in comparison to other methods (Figs. 8 and 9).

In conclusion, the implementation offers new opportunities to simultaneously examine response time and responses. We hope that it will prove to be a useful enrichment to the current modeling landscape.

## Data Availability

All R scripts for the simulations and the empirical analysis, as well as experimental datasets, are available at the Open Science Framework: https://osf.io/486up/. The simulation study was not preregistered.

## Footnotes

1 Vandekerckhove et al. (2011) proposed to implement inter-trial variabilities in a Bayesian framework by using the likelihood function of the basic (four-parameter) diffusion model and adding hyper-distributions for starting point, drift rate and non-decision time. In this framework, the specific parameter values for each trial of an experiment are drawn from the respective hyper-distributions. While this idea is theoretically interesting, this procedure led to convergence problems in our applications and could therefore not be used.

2 The precision value controls the accuracy in the computation of the partial derivatives. The default value for the precision is $10^{-4}$. The user can provide smaller values for increased precision. Note that this precision value only changes the precision in the computation of the partial derivatives, but not of the DM density itself. The precision value for the density is internally fixed to $10^{-6}$ and cannot be changed by the user. The partial derivatives determine the directions in which the parameter space is explored in MCMC sampling. Requesting more accurately computed derivatives may thereby help to increase the efficiency of the exploration of the parameter space, but it trades off against a time cost for computing more accurate derivatives. The validity of the algorithm as such is not influenced by this parameter.

3 Thus, we test whether the test statistic $-2\sum _{i=1}^{18}\log (p_i)$ indicates a violation of the composite hypothesis that the *p*-values themselves stem from a uniform distribution as they should under the $H_0$ of uniformly distributed histograms. Under the $H_0$, the test statistic should follow a $\chi ^2$-distribution with 36 degrees of freedom.

## Code Availability

The code will be available in Stan and is expected to be included into Stan with the next release in the beginning of the year 2023. Until then the implementation is available here: https://github.com/Franzi2114/math/tree/feature/issue-2682-Add-7-parameter-DDM-PDF, and an instruction on how to get started with the new function can be found here: https://github.com/Franzi2114/math_HOW-TO-USE.
